# Toxicological Analysis of Acetamiprid Degradation by the Dominant Strain Md2 and Its Effect on the Soil Microbial Community

**DOI:** 10.3390/toxics12080572

**Published:** 2024-08-05

**Authors:** Jiale Zhang, Xin Wang, Wanlei Yue, Jia Bao, Mengqin Yao, Ling Ge

**Affiliations:** 1 School of Environmental and Chemical Engineering, Shenyang University of Technology, Shenyang 110870, China; 18704120001@139.com (J.Z.); m15222487370@163.com (W.Y.); z1055428700@163.com (L.G.); 2 School of Chemistry and Chemical Engineering, Guizhou University, Guiyang 550025, China; mqyao@gzu.edu.cn

**Keywords:** neonicotinoid insecticide, biodegradation, microbial community, metabolic route

## Abstract

Microbial degradation is acknowledged as a viable and eco-friendly approach for diminishing residues of neonicotinoid insecticides. This study reports the dominant strain of Md2 that degrades acetamiprid was screened from soil and identified as *Aspergillus heterochromaticus*, and the optimal degradation conditions were determined. Research indicated that the degradation of Md2 to 100 mg/L acetamiprid was 55.30%. Toxicological analyses of acetamiprid and its metabolites subsequently revealed that acetamiprid and its metabolites inhibited the germination of cabbage seed, inhibited the growth of *Escherichia coli*, and induced the production of micronuclei in the root tip cells of faba beans. Based on the analysis of metabolic pathways, it has been determined that the primary metabolic routes of acetamiprid include N-demethylation to form IM-2-1 and oxidative cleavage of the cyanoimino group to produce IM-1-3. Using 16S rRNA high-throughput sequencing, the results showed that acetamiprid and Md2 elevated the relative abundance of *Acidithiobacillus*, *Ascomycetes*, and *Stramenobacteria*, with increases of 10~12%, 6%, and 9%, respectively, while reducing the relative abundance of *Acidobacteria*, *Chlorobacteria*, *Ascomycetes*, and *Sporobacteria*, with decreases of 15%, 8%, 32%, and 6%, respectively. The findings will facilitate the safety evaluation of the toxicological properties of neonicotinoid insecticides, their biodegradable metabolites, and associated research on their degradation capabilities.

## 1. Introduction

Acetamiprid, one of the most significant neonicotinoid pesticides, has been extensively advocated and employed in agricultural crop protection globally [1,2]. Acetamiprid targets insect nicotinic (acetylcholine) receptors (nAChR), causing abnormal excitation in pests leading to complete convulsions and paralysis, ultimately resulting in their death [3]. It has been used to control Hemiptera, mainly aphids, tassel-winged insects and Lepidoptera, to protect a wide range of crops, especially vegetables, fruits, and tea [4]. Due to its high insecticidal efficacy and lack of cross-resistance with conventional long-acting insecticides, it has been widely used over the past two decades [5]. Neonicotinoids are efficient insecticides with low toxicity, but their long-term usage has generated extensive environmental problems [6]. Some reports indicate that there is a potential toxicity risk to nontarget organisms (bees, birds, and mammals) [7] and a threat to their survival [8]. Other studies have suggested that acetamiprid may decrease human fertility [9].

Moreover, besides spraying on crop surfaces, root irrigation, and seed treatment, considerable amounts of the pesticide eventually enter the soil [10,11,12]. Extensive application of acetamiprid negatively impacts soil biochemical characteristics and microbial activity [13,14]. It can reduce microbial diversity, alter the community structure of soil microorganisms, affect soil bacterial abundance [15], and significantly inhibit soil respiration and phosphatase activity [16]. These impacts have prompted researchers to actively explore rapid and effective remediation strategies to mitigate these adverse environmental effects. Previous studies have shown that the microbial degradation of neonicotinoids is considered to be the most efficient and environmentally friendly in situ repair pathway [17]. Using potential and degradative microorganisms, which can grow and survive under high-stress concentrations of insecticides, offers a possible opportunity for the remediation of toxic pollutants from contaminated environments [18,19,20,21,22].

To this end, the present study undertook the following specific investigations: Initially, it explored the screening and identification of the dominant strain Md2 from soil that can effectively degrade acetamiprid, and how to screen out strains with efficient degradation ability from a large number of soil microorganisms has been a focus of attention for many researchers and scholars. Secondly, the dominant strain was utilized to degrade 100 mg/L of acetamiprid. The effects of pH, temperature, and inoculum size on the degradation rate were analyzed using the response surface methodology, and the optimal degradation conditions were determined. Subsequently, the toxicological properties of acetamiprid and its metabolites were assessed. The changes in toxicity during the initial and metabolic stages of acetamiprid degradation and their impact on biological systems were determined. Additionally, the metabolic pathways of acetamiprid were analyzed, which not only aids in a deeper understanding of the degradation process but also helps evaluate the potential environmental and biological impacts of its metabolites. Finally, the impact of the acetamiprid-degrading dominant strain Md2 on microbial diversity and community composition in contaminated soil was investigated using Illumina MiSeq high-throughput sequencing technology. This area has received relatively little attention so far. Simulating and assessing the effects of dominant strains on soil ecosystems under experimental conditions is of significant importance for environmental protection and agricultural sustainability. This study comprehensively tracked the process of microbial remediation of pesticide-contaminated soil, aiming to address the critical issue of pesticide residues in soil through the use of dominant strains. The findings are expected to provide scientific information to support the rational use of insecticides.

## 2. Materials and Methods

### 2.1. Chemicals and Materials

Acetamiprid (99%) was purchased from Shandong Zhongnong United Biotechnology, Inc. (Jinan, China). Methanol and acetonitrile (HPLC grade) were purchased from Thermo Fisher Scientific (China), Inc. (Beijing, China). Agarose was purchased from Beijing Aobixing Biotechnology, Inc. (Beijing, China). All other chemicals and reagents were of analytical grade and purchased from China National Medicine Chemical Reagent Inc. (Huaian, China). The composition of Luria-Bertani (LB) liquid medium was as follows: 10 g peptone, 5 g yeast extract, 5 g NaCl at pH 6, and distilled water to 1 L. Solid medium was prepared by incorporating 1.5% agarose into the previously described liquid medium. The inorganic salt medium (L^−1^) comprised 4 g Na_2_HPO_4_, 1.5 g KH_2_PO_4_, 1 g NH_4_Cl, 0.2 g MgSO_4_•7H_2_O, 0.02 g CaCl_2_, 0.03 g FeSO_4_•7H_2_O, 1.0 g NaNO_3_, and 1 mL of trace elements solution at pH 6. Malt extract liquid medium (L^−^^1^) was prepared using 20 g malt extract. All media were sterilized at 121 °C for 30 min.

### 2.2. Enrichment, Isolation, and Characterization of the Acetamiprid-Degrading Strain

In this study, the soil samples used for strain screening were collected from the 5–15 cm surface layer of soil in areas of the campus that had not been previously treated with acetamiprid. All soil samples were taken at five randomly selected points, impurities were removed, and the samples were thoroughly mixed and placed in sealed polyethylene bags. The experimental soil samples were brown soil from the campus that had not been treated with acetamiprid (pH 7.9, organic matter content 1.67%, total salt content 0.11 ms/cm). The surface soil was air-dried in a cool place, gently ground, and sieved through a 40-mesh sieve. An acetamiprid biodegradation system was constructed using five grams of sieved soil and 100 mL of solution containing 20 mg/L acetamiprid at pH 7.0, in which acetamiprid served as the sole source of carbon, nitrogen, and energy. The system was incubated in a rotary shaker at 150 rpm at 30 °C for 7 days [23]. Every 7 days, 10% inoculum was transferred to a fresh medium until the acetamiprid concentration reached 200 mg/L. The 10 mL mixed bacterial solution was subsequently transferred to an inorganic salt medium containing 90 mL 100 mg/L acetamiprid and incubated in a rotary shaker for 7 days. The degradation rate of the formed mixed bacterial consortia was determined using high-performance liquid chromatography (methanol:water = 60:40 (*V*/*V*), column temperature 40 °C, flow rate 1 mL/min) [24]. The bacterial consortia exhibiting superior degradation effects were further isolated and purified. The degradation rate of each individual pure strain was then measured, and the strain with the highest degradation rate was selected to be numbered and preserved for subsequent studies.

The isolated strain was designated Md2 and analyzed through 16S rRNA gene sequencing. Cell morphology was observed under an optical microscope using cells from exponential growth. DNA was extracted using a Genomic DNA Extraction Kit [25], and the universal primers ITS1 (TCCGTAGGTGAACCTGCGG) and ITS4 (TCCTCCGCTTATTGATATGC) were used to amplify the ITS region. PCR products were recovered using an AxyPrepDNA gel Recovery Kit and DNA sequenced. The PCR amplification products were subsequently validated using agarose gel electrophoresis and compared with the NCBI 16S database [26] to obtain the strain with the highest homology to Md2.

### 2.3. Optimization of Acetamiprid-Degrading Conditions

The dominant strain Md2 underwent cultivation in LB liquid medium with 100 mg/L acetamiprid and was incubated on a shaker at 150 rpm for 7 days. To evaluate bacterial proliferation, samples were taken at 12 h intervals, followed by analysis of the cell-free supernatants for acetamiprid degradation [27]. The response surface method (RSM) was used to optimize the degradation conditions of acetamidine by strain Md2. Based on the methodology of Q. Zhai et al., the significant variables influencing the biodegradation of acetamiprid were optimized using the Box–Behnken design. A total of 17 experiments were conducted in a random order [24]. The concentration of acetamiprid was adjusted to 100 mg/L, with the optimal ranges of three factors selected for acetamiprid biodegradation, i.e., pH (6~8), temperature (25~35 °C), and inoculum size (0.1~0.3 g) [24,28,29]. The collected samples were analyzed using HPLC.

### 2.4. Toxicity Analysis of Acetamiprid and Its Metabolites

#### 2.4.1. Effects of Acetamiprid and Its Metabolites on Cabbage Seed

Seed germination experiments serve as an excellent indicator for assessing the impact of pollutants [30]. The experiment concentrated on cabbage seeds to analyze the toxicity of acetamiprid and the toxicity of its metabolites within 14 days of degradation by the dominant strain. The Petri dishes were wrapped in newspaper and sterilized with steam at 121 °C for 30 min. Under aseptic conditions, UV-sterilized filter paper was cut and placed into the Petri dishes. Different treated inorganic salt media were evenly applied to moisten the filter paper. The top and bottom of the Petri dishes were lined with a thin layer of paper, and the corresponding solutions were sprayed to maintain humidity. The experiment involved three treatment groups: (A) sterilized (121 °C 30 min) inorganic salt medium with the addition of 5 mL acetamiprid (concentrations of 5 mg/L, 10 mg/L, 20 mg/L, 50 mg/L, and 100 mg/L); (B) sterilized inorganic salt medium supplemented with its corresponding concentration of metabolites; (C) blank control group CK (sterilized inorganic salt medium). Three parallel experiments were conducted for each group. A specific amount of cabbage seeds was evenly scattered in the Petri dishes and subsequently stored in a steady-temperature incubator at 25 °C in darkness. Samples were taken at 24 h intervals, with 5 days as the testing cycle. The seed germination rate was determined by counting the number of germinated seeds, and germination shoot length was measured.

#### 2.4.2. Effects of Acetamiprid and Its Metabolites on *Escherichia coli*

*Escherichia coli* is frequently used as a classic model organism in pesticide toxicology studies due to its ease of cultivation and manipulation, as well as its rapid growth cycle [31]. Therefore, *Escherichia coli* was utilized to evaluate the toxicity of acetamiprid (before/after) biodegradation by the dominant strain in this study. A small amount of preserved *E. coli* was selected, inoculated into LB solid medium, and incubated at 37 °C and 150 rpm for 2 days. After three rounds of subculturing, the bacteria were inoculated into LB liquid medium and incubated at a constant temperature for 18 h to prepare the activated *E. coli* stock solution [32]. The experiment involved three treatment groups: to freshly prepared sterilized LB liquid medium, (A) add 10 mL inorganic salt solution of the dominant strain degrading acetamiprid (100 mg/L) for 14 d and 1 mL activated *E. coli* stock solution with OD600 value of 0.5; (B) add 10 mL sterilized inorganic salt solution containing 100 mg/L acetamiprid and 1 mL of identical *E. coli* stock solution; (C) blank control group CK, 10 mL of sterilized inorganic salt solution and 1 mL of identical *E. coli* stock solution. Throughout the experiment, the absorbance at OD600 of the *E. coli* solution was measured at intervals (2 h, 4 h, 6 h, 8 h, 10 h, and 12 h) using a UV-visible spectrophotometer to monitor *E. coli* growth. Three parallel experiments were conducted for each group.

#### 2.4.3. Micronucleus Test

Faba bean seeds of uniform size were washed and placed in a 25 °C light-proof incubator. They were immersed in deionized water for 36 h, with the water changed every 12 h. The fully soaked seeds were then placed in a dissecting tray lined with moist absorbent cotton and gauze. The seeds were germinated in the dark at 25 °C for 12–24 h. When the embryonic roots reached a length of 1.5~2 cm, each Petri dish received four seeds, which were then exposed to acetamiprid solution in varying concentrations of 5 mg/L, 10 mg/L, 20 mg/L, 50 mg/L, and 100 mg/L for 5 h [33]. The metabolite treatment solution post-degradation of acetamiprid by the dominant strain at a corresponding concentration was used as a control experiment. Post-treatment, the seeds and root tips underwent a triple rinse with deionized water and were incubated in a constant-temperature chamber at 25 °C for 24 h, aiding in the repair and growth of root tip cells. Subsequently, the cut 1 cm long root tips were fixed in 2 mL Carnot’s fixative for 36~48 h. Rinsing with SO_2_ solution and deionized water resulted after the Feulgen staining process. Slides were fabricated by the pressing method, and three root tip cells removed from each treatment described above were visualized under a light microscope, with 1000 cells counted per root tip. Ultimately, the process involves identifying micronuclei, counting MCN frequencies per 100 tetrads (MCN‰), numbering mitotic cells, and calculating the mitotic index (MI). The results were obtained and expressed as mean ± standard error.

### 2.5. Bioremediation of Acetamiprid-Contaminated Soil by Dominant Strain Md2

To investigate the degradation ability of Md2 on acetamiprid in simulated contaminated soil, 20 mg of acetamiprid was dissolved in 50 mL of methanol to prepare a 400 mg/L acetamiprid–methanol solution. An amount of 50 mL of this solution was evenly sprayed onto 1 kg of naturally air-dried soil, mixed thoroughly, and dried in a dark fume hood for 24 h until the methanol evaporated. This process yields simulated contaminated soil with an acetamiprid concentration of 20 mg/kg. An amount of 0.1 g of Md2 was separately inoculated into sterilized (121 °C 30 min) and non-sterilized soil samples through the plate punching method, ensuring thorough mixing and dispensing into small flowerpots. The experiment included four treatment groups: (A) W-D: non-sterilized soil with only acetamiprid added; (B) M-D: sterilized soil with only acetamiprid added; (C) W-DJ: non-sterilized soil with Md2 inoculated and acetamiprid added; (D) M-DJ: sterilized soil with Md2 inoculated and acetamiprid added. Subsequently, the soil was placed in a constant-temperature incubator with the cultivation conditions: a 12 h day cycle (25 °C, light intensity of 40 lx) and a 12 h night cycle (20 °C, no light). Throughout the study, sterile water was regularly added to maintain the soil moisture at 20% of its water-holding capacity [34]. At specific intervals (0, 3, 5, 7, 10, and 14 days), 5 g of cultured soil samples were obtained to measure the degradation rate, with three parallel experiments per group.

### 2.6. Analysis of Microbial Communities by High-Throughput Sequencing

DNA extraction from soil samples was performed using the FastDNA Spin Kit (MP Biomedicals, Santa Ana, CA, USA). Validation of the isolated DNA was conducted through 0.8% agarose gel electrophoresis, while the quality of DNA was evaluated using a UV spectrophotometer. DNA samples were preserved at −20 °C to facilitate further PCR amplification. For PCR amplification, the fungal ITS region of the 16S rRNA gene was amplified using universal primers TCCGTAGGTGAACCTGCGG and TCCTCCGCTTATTGATATGC. The PCR cycling procedure entailed an initial denaturation at 98 °C for 30 s, followed by 27 cycles of 15 s at 98 °C, 30 s at 50 °C, and 30 s at 72 °C, and a final extension at 72 °C for 5 min [35]. The PCR products underwent purification using a 2% agarose gel DNA Purification Kit before being dispatched to PacBio in Shanghai, China, for sequencing.

Community DNA segments underwent bipartite sequencing utilizing the Illumina MiSeq system. The paired-end sequences were merged using FLASH (version 1.2.11, https://ccb.jhu.edu, accessed on 23 January 2023) and assigned to each sample based on unique barcodes and primers using Mothur software (version 1.35.1, http://www.mothur.org, accessed on 27 February 2023). Sequence analysis was performed using USEARCH software (version 8.0.1517, http://www.drive5.com/usearch/, accessed on 27 February 2023). Based on ≥97% similarity, sequences were assigned to the same operational classification unit (OTU). OTUs were categorized using the RDP classifier (http://rdp.cme.msu.edu/, accessed on 27 February 2023) with 80% confidence to obtain species-level taxonomy information for each OTU. Subsequent analysis of α-diversity and β-diversity was performed using QIIME (version 1.9.1) and R software (version 2.15.3) based on the normalized data output [23]. Community diversity was estimated using the Chao1, observed species, Shannon, and Simpson indices. Principal coordinates analysis (PCoA) was used to visualize intergroup distances and correlations in bacterial community structure.

## 3. Results and Discussion

### 3.1. Identification of Acetamiprid Degrading Strain

**Figure 1:**
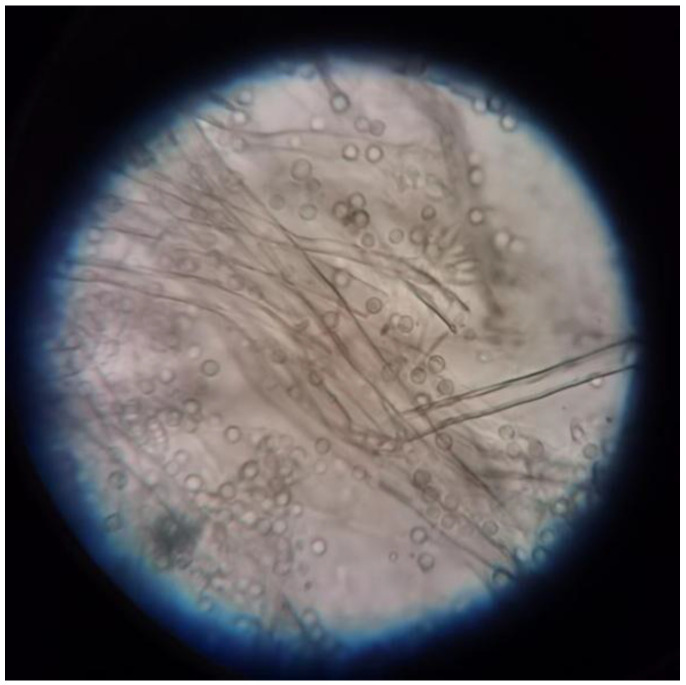
Optical microscope image of strain Md2 on malt powder culture medium (×400).

The dominant strains Md2 that utilized acetamiprid as the sole carbon and nitrogen source were isolated from vegetation soil. The fungus Md2 is distinguished by its radial peduncles of conidiophores that are colorless or mildly yellowish, featuring green spherical conidiophores (Figure 1). Culturing in malt extract medium, Md2 appeared fluffy and initially white, turning pale green after 1~2 days and subsequently turning to ash green within 3~5 days. Certain colonies formed droplets that were either colorless or yellowish, while the reverse side of the colony exhibited a light-yellow hue. Sequencing of the 16S rRNA gene revealed 99.63% sequence identity with *Aspergillus versicolor*. Based on its morphology and genetic sequencing data, Md2 was identified as *Aspergillus versicolor*.

### 3.2. Optimization of Acetamiprid Degrading Conditions

**Figure 2:**
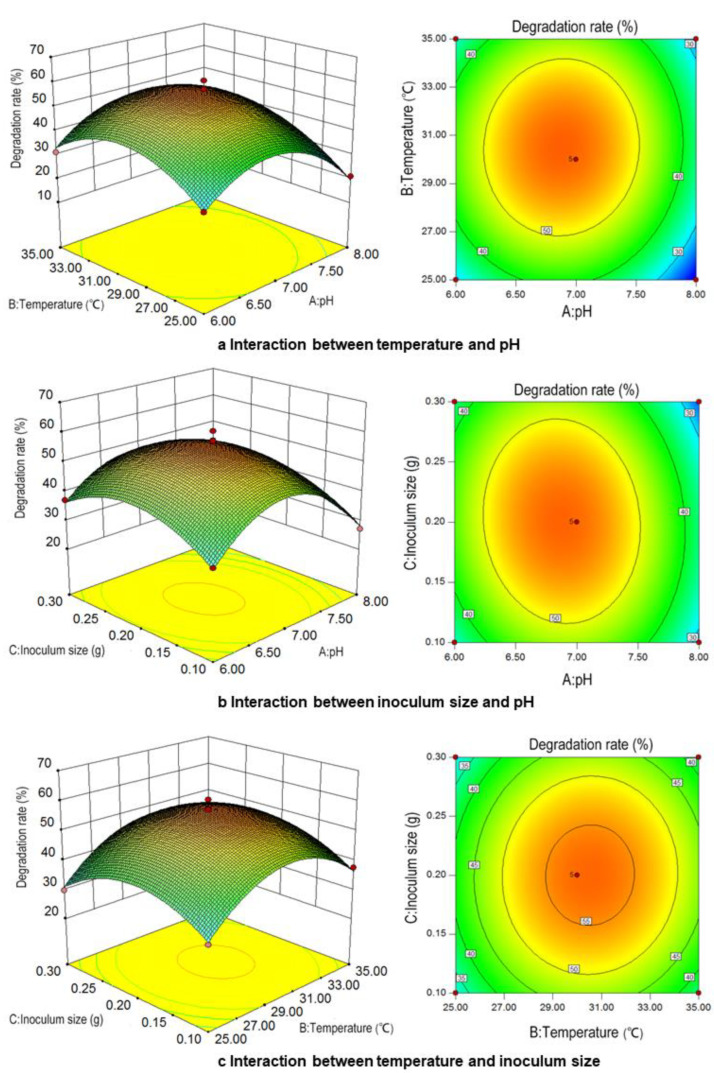
Response surface diagram of culture condition interaction with degradation of acetamiprid by Md2.

A multiple linear regression analysis was applied to the experimental data for three factors (pH (*X*_1_), temperature (*X*_2_), and inoculum size (*X*_3_)). Each factor varied at three levels (−1, 0, and 1). A polynomial quadratic equation was found to represent the percentage of acetamiprid degradation, as given by the following mathematical expression:

$$Y=56.58-4.09X_{1}+2.72X_{2}-0.047X_{3}+0.92{X_{1}X}_{2}-1.65{X_{1}X}_{3}+0.50X_{2}X_{3}-16.58{\left(X_{1}\right)}^{2}-12.87{\left(X_{2}\right)}^{2}-9.50{\left(X_{3}\right)}^{2}$$

where *Y* is the degradation rate of Md2 to acetamiprid, the regression coefficient R^2^ = 0.9824, and *p* < 0.0001, indicating the accuracy of the employed model and good prediction of the response. The *p*-values for the three factors were as follows: pH, *p* = 0.0031; temperature, *p* = 0.0220; and inoculum size, *p* = 0.9606. Among these, influence size: pH > temperature > inoculum size. Through the analysis of contour plots, it was determined that the optimal degradation rate of acetamiprid is observed at intermediate values of pH, temperature, and inoculum size (Figure 2). The results indicated that the optimal conditions for Md2 to degrade acetamiprid were pH 6.8, a temperature of 30 °C, and an inoculum size of 0.2 g. According to the model fit, 55.30% of the acetamiprid degradation rate of 100 mg/L by Md2 within 7 days was obtained, which closely matches the predicted value of 56.96%, demonstrating the robustness of the response surface model in fitting the experimental data.

### 3.3. Biotoxicity Analysis of Acetamiprid and Its Metabolites

#### 3.3.1. Effects of Toxicity on Cabbage Seeds

**Figure 3:**
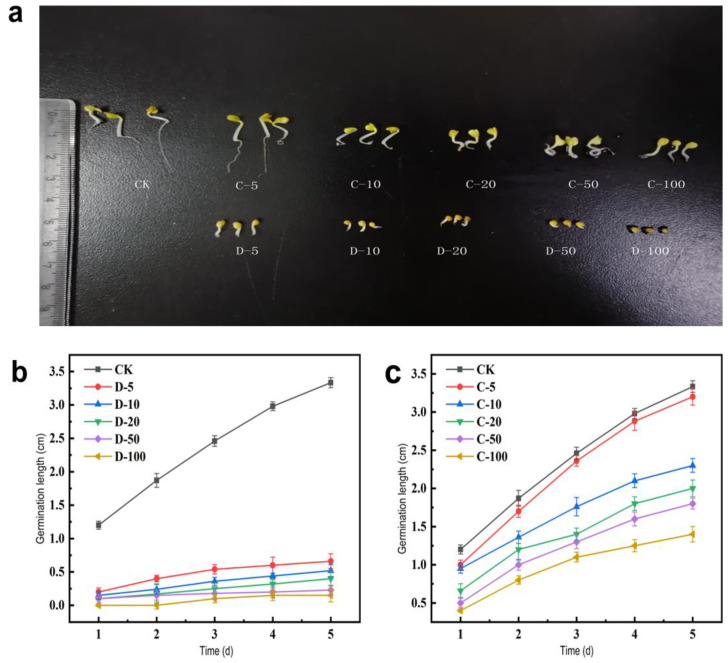
(**a**) Effect of acetamiprid and its metabolites on seed germination of cabbage; effects of acetamiprid (**b**) and its metabolites (**c**) on the germination length of cabbage seeds.

This study examines the impact of acetamiprid on the germination and seedling growth of cabbage to evaluate its toxicity to crops. Similarly, H. Qi et al. explored the impact of neonicotinoids on the growth of cabbage. This experiment found that acetamiprid and its metabolites can inhibit the seed germination and germination length of cabbage (Figure 3), with acetamiprid showing a more significant effect, particularly at a concentration of 100 mg/L, significantly inhibited seed sprouting. Previous studies have found that several pesticides, including acetamiprid, alpha-cypermethrin, and pyriproxyfen, inhibit seed germination in various plants to varying degrees [24]. Such suppression happens because specific concentrations of acetamiprid and various pesticides hinder seed respiration, affecting the germination process and resulting in inhibited seed germination, thereby causing plant seeds to suffer from phytotoxicity [30]. Compared to the metabolite group, the seed germination rate in the acetamiprid group was 18.00% to 28.66% lower, indicating that the biotoxicity of the metabolites is lower than that of acetamiprid. Examining the germination length within 5 days (Figure 3b,c) indicates that germination length decreases with increasing concentrations of acetamiprid and its metabolites, while the seedling length in the group treated with 5 mg/L of metabolites was similar to that of the control group. These results suggest that pesticides and products of their metabolism can potentially accumulate and have additive and chronic effects [36,37]. In addition, the results find that the toxicity of acetamiprid was reduced after biodegradation by the dominant strain Md2, decreasing environmental residues and providing an eco-friendly solution for soil remediation.

#### 3.3.2. Effects of Toxicity on *E. coli*

**Figure 4:**
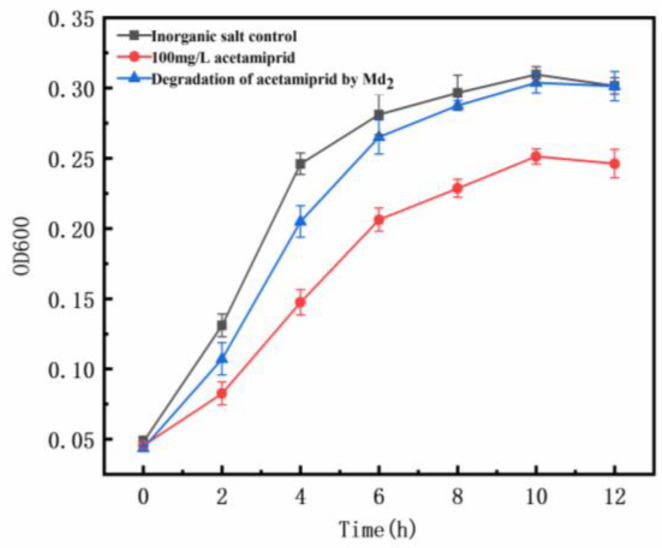
Effects of acetamiprid and its metabolites on *Escherichia coli*.

The toxicity analysis of *Escherichia coli* (*E. coli*) was conducted over a 0–12 h period, with measurements taken every 2 h to evaluate the physiological impact of acetamiprid on bacterial growth. Figure 4 illustrates that introducing 100 mg/L acetamiprid significantly inhibits the growth rate of *E. coli*, corroborating findings by Saurabh Bhatti et al. [32]. Furthermore, the metabolite group shows some initial inhibition of *E. coli* growth within the first 6 h, followed by a diminishing inhibitory effect, with growth conditions approaching those of the blank control group after 10 h. These results indicate that acetamiprid decreases the growth rate and activity of *E. coli*, significantly suppressing bacterial growth. However, the metabolites resulting from degradation by the dominant strain Md2 exhibit a transient inhibitory effect on *E. coli* growth within a certain timeframe, with their toxicity significantly lower than that of acetamiprid. This phenomenon may be attributed to the toxic effects of acetamiprid or inhibitory intermediate metabolites on the growth metabolism of *E. coli*, thereby inhibiting its growth. This will provide a theoretical and practical foundation for future risk assessments of acetamiprid’s impact on biological systems.

#### 3.3.3. Micronucleus Test

Utilizing the micronucleus test, the degree of DNA damage in faba bean root tip cells was assessed to predict the genetic toxicity caused by acetamiprid and its metabolites, as demonstrated by MCN‰ and MI (Table 1). The study observed root tip cell micronuclei using an optical microscope (Nikon YS2 Alphaphot, Tokyo, Japan) at 400 times magnification. Results from the study indicate that the MCN‰ and MI of faba bean root tip cells increase with higher concentrations of acetamiprid and its metabolites. Similar findings by Li Wenyi et al. [33] demonstrated an increase in MCN‰ with increasing exposure time of faba beans to the same concentration of acetamiprid at 18, 24, and 48 h. These results suggest that acetamiprid may cause damage to cell chromosomes, resulting in the formation of more micronuclei during cell division. Conversely, the metabolite group exhibited significantly lower MCN‰ and MI. The experimental results show that faba bean root tip cells were hypersensitive to acetamiprid-induced micronuclei. This effect is attributed to the strong mutagenic effect of acetamiprid on the root tip cells of faba bean seeds, which interferes with the division of the spindle in mitotic cells in the plant, thus affecting cell division [38]. Furthermore, concentrations exceeding a certain threshold of acetamiprid impair the repair ability of the cell itself, causing the spindle apparatus to lag and remain in the cytoplasm as micronuclei. Recent research has established that pesticides not only act as chromosome breakers but also disrupt spindle apparatus function [39]. Therefore, acetamiprid induces micronuclei formation in faba bean root tip cells, causing severe damage to chromosome and gene structures, with greater concentrations resulting in more significant genetic damage. Importantly, the results confirm that the dominant strain Md2 effectively mitigates the toxicity of acetamiprid.

### 3.4. Analysis of the Degradation Mechanism of Acetamiprid

**Figure 5:**
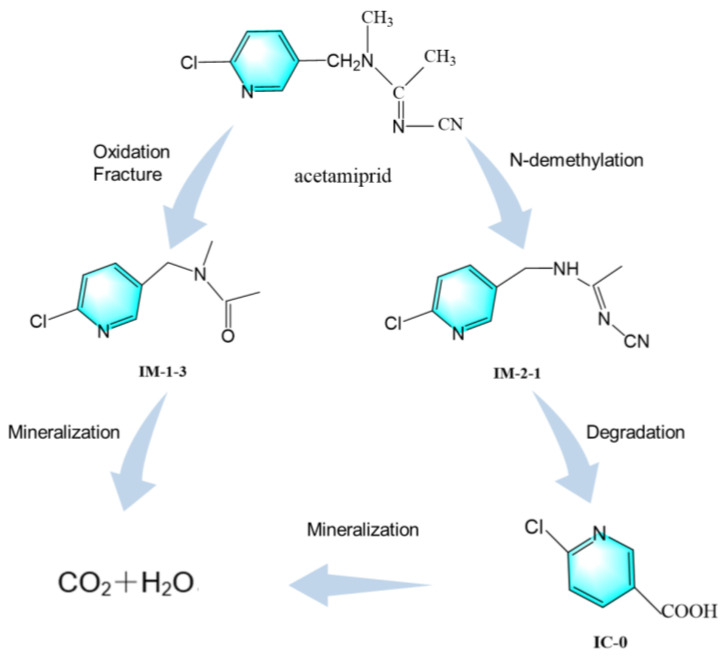
There may be degradation products in acetamiprid.

Acetamiprid ((E)-N^1^-[(6-chloro-3-pyridyl)methyl]-N^2^-cyano-N^1^-methylacetamidine, C_10_H_11_ClN_4_, AAP) is a neonicotinoid insecticide. Figure 5 shows the possible major metabolic pathways of acetamidine. In this study, the main metabolic pathway of acetamiprid is the oxidative cleavage of the pharmacophore -N=C-CN of AAP by Aspergillus versicolor Md2 to produce IM-1-3 (N-[(6-chloropyridin-3-pyridyl)methyl]-N-methylacetamide). This intermediate undergoes further oxidation steps and ultimately mineralizes into CO_2_ and H_2_O. At the same time, *Aspergillus versicolor* Md2 demethylates AAP to produce N-demethylated acetamiprid IM-2-1 (N^1^-[(6-chloro-3-pyridyl)methyl]-N^2^-cyanoacetamidine); the procedure is related to the cytochrome P450 [40]. After demethylation, IM-2-1 is further degraded to IC-0 (6-chloronicotinic acid), which is eventually mineralized to CO_2_ and H_2_O. The main metabolic pathway for the degradation of AAP by *Aspergillus versicolor* Md2 is the production of IM-1-3 and IM-2-1, which are one magnitude less biologically active than AAP [40]. IM-1-3 are almost biologically inactive owing to the lack of the pharmacophore -N=C-CN [41]. Therefore, the metabolism of AAP by *Aspergillus versicolor* Md2 is a process with decreased biotoxicity.

### 3.5. Effect of Md2 on the Degradation of Acetamiprid in Soil and Microbial Community Diversity

Pesticides permeate the soil through methods such as spraying, root irrigation, and seed coating. Although these chemicals are not specifically intended for the soil, the soil ecosystem quietly endures their repercussions, prompting soil microorganisms to enact a range of responses against external interference. These responses include changes in microbial diversity and community structure, the proliferation of favored populations, and even the gradual decline of particular microbial communities that are incapable of withstanding the exposure.

#### 3.5.1. Degradation of Acetamiprid by Dominate Strain Md2

**Figure 6:**
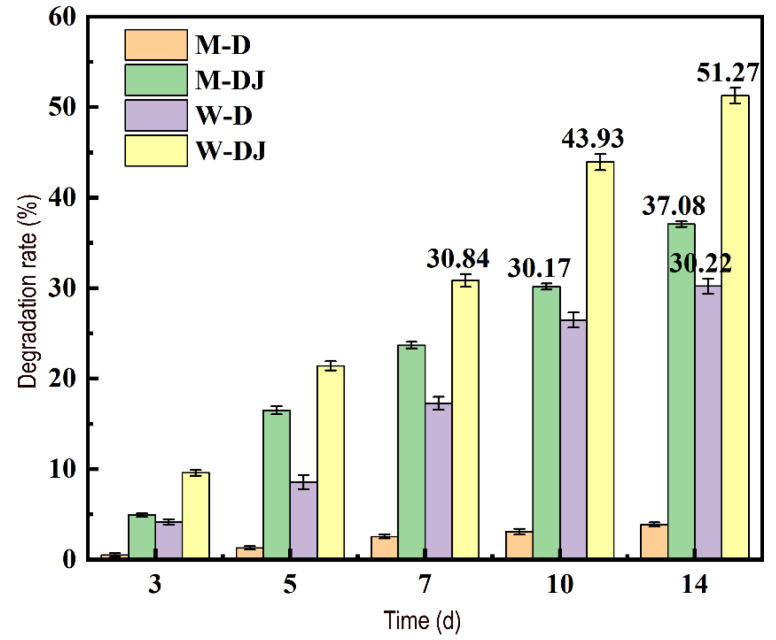
Degradation rate of acetamiprid in soil samples under different treatments.

The degradation ability of Md2 towards acetamiprid was further evaluated based on the residual content of acetamiprid in sterilized and unsterilized soils treated with Md2 (a comparison of average degradation rates is shown in Figure 6). On the third day, the degradation rate of Md2 was less than 10%, possibly because excessive concentrations of pesticides may cause toxic effects [42] and decrease degradation capability. However, the degradation rate of Md2 exhibited a gradual increase with time. This suggests that the dominant strain Md2 gradually adapted to the soil environment containing acetamiprid. The unsterilized soil with added Md2 exhibited stronger degradation capability, reaching the highest degradation rate of 51.27% by the 14th day. This demonstrates that Md2 can proliferate steadily in soil, a result further corroborated by fungal community composition analysis. Furthermore, higher degradation of acetamiprid by Md2 was observed in unsterilized soil (51.27%) compared to sterilized soil (37.08%), which may be due to the presence of other types of natural acetamiprid-degrading microorganisms in the soil that metabolize acetamiprid synergistically, thus aiding in soil remediation [43].

#### 3.5.2. Alpha Diversity Index Analysis

**Figure 7:**
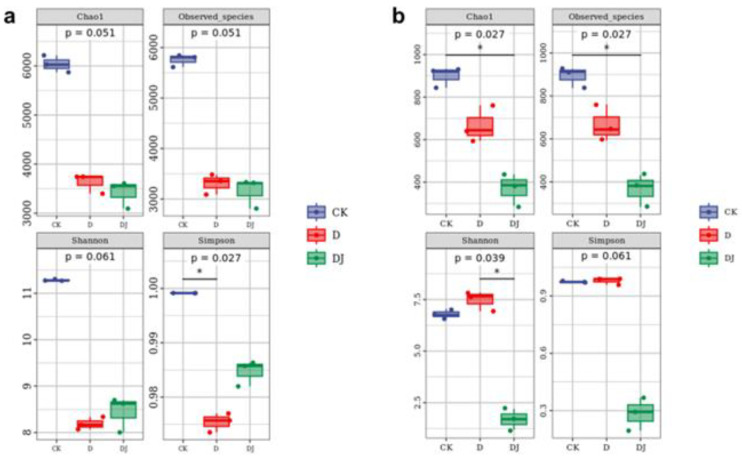
Plot of alpha diversity index of (**a**) bacteria and (**b**) fungi in soil samples supplemented with acetamiprid and dominant strains.

Alpha diversity was used to evaluate changes in bacterial and fungal diversity in soil communities (Table 2). Compared to the CK, the alpha diversity index of the soil decreased considerably under the influence of acetamiprid and its metabolites, demonstrating that acetamiprid resulted in a reduction in the number, variety, and diversity of bacterial and fungal species in the community. In the bacterial community (Figure 7a), the Chao1 and observed species indices notably decreased in the acetamiprid treatment group, indicating a reduction in bacterial community abundance. In the fungal community (Figure 7b), the alpha diversity indices decreased with acetamiprid treatment, indicating a reduction in fungal community abundance and diversity. However, the Shannon and Simpson indices of the group after being treated with Md2 were restored to levels similar to CK, suggesting that Md2 may partially degrade acetamiprid, leading to significant microbial diversity recovery. From the experimental results, it emerges that acetamiprid significantly diminished the alpha diversity of bacteria and fungi in soil samples. This might be related to some potentially degrading bacteria in the soil affecting acetamiprid [44]; such bacteria are capable of utilizing acetamiprid as a substrate, which could induce an increase in their population size in soils, thereby intensifying competitive selection among microorganisms [45], resulting in a decrease in alpha diversity indices.

#### 3.5.3. Beta Diversity Index Analysis

**Figure 8:**
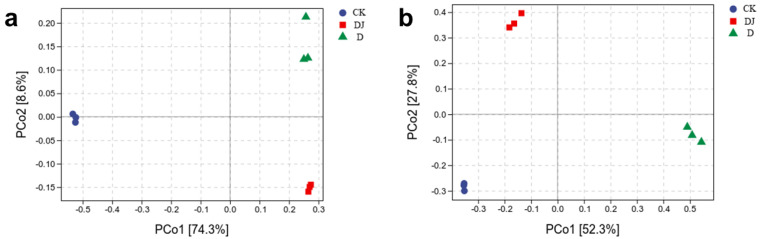
Differences in soil microbial (**a**) bacterial, (**b**) fungal community structure between the addition of acetamiprid and the dominant strains based on PCoA analysis.

Beta diversity is represented by principal coordinates analysis (PCoA) to illustrate microbial community differences among different sample groups. The PCoA results (Figure 8) show that the three replicate samples of soil samples from the control group (CK) and two experimental groups (D and DJ) cluster in different quadrants. In bacterial samples (Figure 8a), the first principal component PCo1 (74.3%) has a greater impact on separation than the second principal component PCo2 (8.6%). The sampling points of the two experimental groups and the control group are distinctly separated, indicating significant changes in soil bacterial community composition influenced by acetamiprid and the dominant strain Md2. In fungal samples (Figure 8b), the first principal component PCo1 (52.3%) has a greater impact on separation than the second principal component PCo2 (27.8%). The distance between CK and DJ is significantly smaller than the distance between CK and D, suggesting that fungal community composition underwent significant changes with the invasion of acetamiprid and subsequent addition of Md2, which partially degraded the acetamiprid, resulting in a restoration of stability in fungal community composition over time. These results are further supported by the community composition at the phylum level.

#### 3.5.4. Effects of Dominant Strains and Acetamiprid on the Composition of Microbial Communities

**Figure 9:**
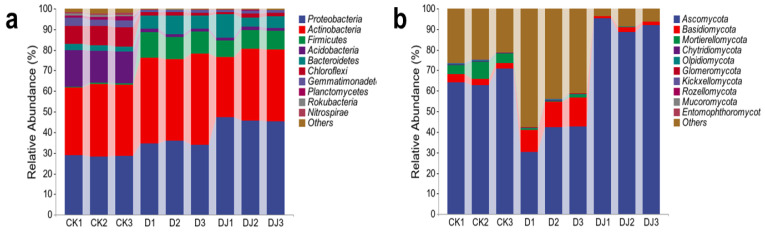
Community composition of (**a**) bacteria and (**b**) fungi at the phylum taxonomic level in soil supplemented with acetamiprid and dominant strains.

At the phylum level, the soil samples from the control group (CK) and experimental groups (D and DJ) exhibited high similarity in bacterial community composition, as depicted in Figure 9. The dominant bacterial phyla detected included *Actinobacteriota* (34.75~41.51%), *Proteobacteria* (28.14~29.02%), and *Acidobacteria* (15.37~17.80%). Treatment with acetamiprid significantly increased the relative abundance of *Firmicutes* (10~12%), *Proteobacteria* (about 6%), and *Bacteroidetes* (4~6%). Conversely, acetamiprid markedly decreased the relative abundance of *Acidobacteria* (about 15%) and *Chloroflexi* (about 8%). Studies suggest that *Firmicutes*, *Proteobacteria*, and *Bacteroidetes* are the main degrading strains of petroleum, polycyclic aromatic hydrocarbons, and pesticides [46,47,48]. This suggests that they may be involved in the biodegradation of acetamiprid, with enhanced metabolic activity and a significant increase in bacterial numbers [49]. The decrease in the relative abundance of certain phyla indicates that their activity was inhibited by neonicotinoid exposure, particularly at high doses. *Proteobacteria* are considered to be a syntrophic taxon, while *Acidobacteria* are regarded as an oligotrophic taxon [50,51], explaining their inverse relationship.

In terms of fungi, the predominant phyla detected were *Ascomycota* (62.72~70.99%) and *Mortierellomycota* (4.5~8.38%). The relative abundance of *Basidiomycota* in soil samples treated with acetamiprid increased significantly (by about 9%). This may be because *Basidiomycetes* can degrade refractory organic matter [52] and thus increase their abundance. The relative abundance of *Ascomycota* (about 32%) and *Mortierellomycota* (about 6%) decreased. In soil samples spiked with acetamiprid-dominant degrading bacteria, the relative abundance of *Ascomycota* reached 91.87%. This is because the dominant degrading bacterium of acetamiprid is *Aspergillus versicolor*, which taxonomically belongs to *Ascomycota*. Results showed that the addition of Md2 had an ameliorative effect on changing the relative abundance of dominant soil phyla due to acetamiprid application, overcoming competition with indigenous microorganisms, and colonizing the contaminated soil successfully. Ultimately, this improves and strengthens the overall fungal community structure of the whole soil [53].

**Figure 10:**
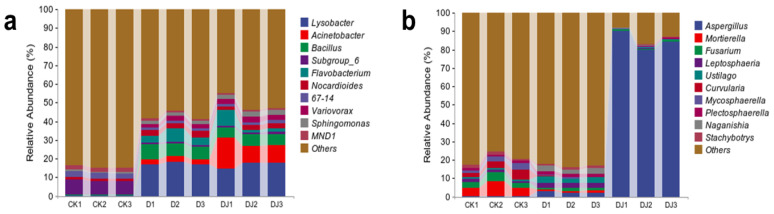
Community composition of soil microorganisms (**a**) bacteria, (**b**) fungi supplemented with acetamiprid and dominant strains at genus taxonomic level.

At the genus level, the differences observed in soil samples treated with acetamiprid and Md2 were more pronounced than at the phylum level (Figure 10). Acetamiprid treatment increased the relative abundance of bacteria such as Lysobacter, Acinetobacter, Flavobacterium, Variovorax, and Sphingomonas while decreasing the relative abundance of Subgroup_6. Lysobacter and Acinetobacter are bacteria commonly associated with plant root symbiosis, and acetamiprid application may inhibit certain symbiotic microbes related to plant roots, allowing Lysobacter and Acinetobacter to gain a competitive advantage in resource competition. Flavobacterium is known to degrade various pesticides including acetamiprid [54], Variovorax are key degraders of pesticides and petroleum pollutants [55,56,57], and Sphingomonas is a versatile degrader capable of utilizing various organic compounds for biodegradation [44]. This suggests their potential involvement in acetamiprid degradation in polluted soil, utilizing pesticides as carbon or energy sources, thus increasing their relative abundance. Comparing D to DJ, the relative abundance of Variovorax and Sphingomonas in DJ was higher, likely due to a symbiotic relationship between Md2 and these bacteria in the soil, synergistically degrading acetamiprid.

At the genus level for fungi, Aspergillus, Mortierellomycota, Fusarium, and Mycosphaerella were detected, as well as other fungi. Mortierellomycota, Fusarium, and Mycosphaerella decreased in relative abundance in D treatment soil samples, but Fusarium recovered to a certain level after Md2 addition. Fusarium and Mycosphaerella play crucial roles in decomposing organic matter in soil, and their functions may be inhibited by acetamiprid use. In DJ treatment soil samples, Aspergillus reached a relative abundance as high as 84.76%, likely due to Md2 belonging to the Aspergillus genus. This significant shift in soil fungal community structure following the addition of dominant bacterial strains in acetamiprid-contaminated soil suggests that with rapid proliferation of certain dominant fungi, eventually the soil community can return to a steady state within a certain period [49]. These results highlight the important role of dominant strain Md2 in mitigating the toxic effects of acetamiprid on soil microbial community composition and demonstrate the restorative effect of Md2 on changing the relative abundance of dominant phyla and genera affected by acetamiprid.

## 4. Conclusions

This study provides new insights into the biodegradation of acetamiprid. The *Aspergillus versicolor* Md2, isolated from soil, achieved a degradation rate of up to 55.30% under optimal conditions (pH 6.8, a temperature of 30 °C, and an inoculum size of 0.2 g) to 100 mg/L of acetamidine. The toxicological properties of acetamiprid and its metabolites were investigated. Acetamiprid and its metabolites inhibited the germination and growth of cabbage seeds, exerted toxic effects on the growth of *E. coli*, and induced the formation of more micronuclei during the cell division of fava bean root tip cells. Additionally, the metabolites of acetamiprid were analyzed, revealing that its primary metabolic pathways are N-demethylation to form IM-2-1 and oxidative cleavage of the cyanoimino group to form IM-1-3. The toxicity of the metabolites was found to be lower than that of the parent compound.

Furthermore, the introduction of the acetamiprid-degrading dominant strain increased the relative abundance of certain phyla (such as *Firmicutes*, *Proteobacteria*, *Bacteroidetes*, and *Basidiomycota*) and genera (such as *Lysobacter*, *Acinetobacter*, and *Flavobacterium*) in the soil, possibly due to their involvement in the biodegradation of acetamiprid as a carbon and nitrogen source. This introduction also decreased the relative abundance of some other phyla (such as *Acidobacteria*, *Chloroflexi*, and *Ascomycota*) and genera (such as *Subgroup_6*, *Mortierellomycota*, *Fusarium*, and *Mycosphaerella*) in the soil. The dominant strain Md2 can effectively reduce acetamiprid residues in the environment, decrease the toxicity of the pesticide and its metabolites, and gradually adapt to contaminated soil, successfully colonizing it, thus contributing to the recovery of dominant phyla and genera in the soil microbiome.

## Figures and Tables

**Table 1:** MCN‰ and MI in faba root tip cells induced by acetamiprid and metabolites.

Concentration (mg/L)	MCN‰ of Acetamiprid Group	MI of Acetamiprid Group	MCN‰ of Metabolite Group	MI of Metabolite Group
0	0.14 ± 0.14 f	0.20 ± 0.12 f	0.20 ± 0.11 f	0.15 ± 0.15 f
5	4.50 ± 0.40 e	4.82 ± 0.58 e	0.94 ± 0.14 e	1.23 ± 0.11 e
10	5.76 ± 0.40 d	8.34 ± 0.43 d	1.64 ± 0.11 d	2.16 ± 0.12 d
20	11.09 ± 0.22 c	10.84 ± 0.51 c	3.03 ± 0.29 c	3.15 ± 0.25 c
50	13.24 ± 0.31 b	13.61 ± 0.73 b	3.85 ± 0.11 b	4.45 ± 0.22 b
100	15.99 ± 0.25 a	16.28 ± 1.05 a	4.68 ± 0.40 a	5.81 ± 0.43 a

The data in the table are the mean ± standard error. The six different letters a, b, c, d, e, and f indicate significant differences between these six groups in the statistical process.

**Table 2:** Index table of alpha diversity of bacteria and fungi in soil samples supplemented with acetamiprid and dominant strains.

	Group	Chao1	Observed-Species	Shannon	Simpson
Bacteria	CK	6038.21	4978.20	11.28	0.99
	D	3626.98	3126.33	8.19	0.98
	DJ	3417.11	3884.23	8.44	0.98
Fungi	CK	899.07	891.87	6.8	0.97
	D	369.96	365.83	1.7	0.28
	DJ	666.96	666.13	7.47	0.98

## Data Availability

The data presented in this study are available on request from the corresponding author.
